# Effects of Positional Traction Integrated With Mobilization With Movement on Cervical Facet Joint Syndrome

**DOI:** 10.7759/cureus.88276

**Published:** 2025-07-19

**Authors:** Apurva Saptale, Siddhi Patrekar, Sawani Aphale, Sandeep Shinde

**Affiliations:** 1 Department of Musculoskeletal Sciences, Krishna College of Physiotherapy, Krishna Vishwa Vidyapeeth (Deemed to be University), Karad, IND

**Keywords:** neck pain, physiotherapy, range of motion, rehabilitation, traction

## Abstract

Background

Cervical facet joint syndrome (CFJS) is a common source of neck pain and disability, often limiting functional activities. While conventional physiotherapy provides symptomatic relief, it may not always ensure optimal recovery. Positional traction, when combined with mobilization with movement (MWM), is a promising strategy aimed at enhancing cervical mobility and reducing pain.

Objectives

This study evaluated the effectiveness of combining positional traction with MWM in reducing pain, improving function, and increasing cervical range of motion (ROM) in individuals with CFJS.

Methods

A comparative study was conducted involving 114 participants diagnosed with CFJS. Participants were randomly allocated into two groups using the envelope method. Group A (control) received conventional physiotherapy, while group B (experimental) received positional traction with MWM alongside conventional treatment. Pain, disability, and ROM were assessed using the visual analog scale (VAS), Neck Disability Index (NDI), and goniometric ROM evaluations, respectively. Data were analyzed pre- and post intervention using SPSS version 26.0 (IBM Corp., Armonk, NY).

Results

Both groups showed significant improvement in all outcomes. The pain scores of group A decreased from 4.98 ± 0.61 to 2.49 ± 0.78 at rest and from 6.34 ± 0.46 to 3.04 ± 1.03 on activity. Group B showed a reduction from 4.83 ± 0.94 to 2.09 ± 0.76 at rest and from 6.09 ± 0.57 to 2.58 ± 0.85 on activity (p-value < 0.0001). However, group B exhibited greater improvements across all outcomes (p-value <0.0001). Post-intervention values revealed enhanced cervical mobility and a more pronounced reduction in pain and functional limitations in the experimental group.

Conclusion

Integrating positional traction with MWM into conventional physiotherapy yields superior clinical outcomes for CFJS patients. This combined approach effectively reduces pain and disability while improving cervical mobility and can be considered a valuable addition to rehabilitation programs.

## Introduction

The phrase “Facet Syndrome” was coined by Ghromley in 1933 [1]. Facet joint pain (facet syndrome) refers to pain originating from the facet joints (zygapophyseal joints or Z joints) at the cervical spine level. It arises from a disruption in normal architecture or function [2]. Neck pain is defined as discomfort or pain localized between the occipital region and the first thoracic vertebra. When this pain extends to surrounding anatomical areas, it is classified as radiating neck pain. In approximately 2% to 11% of cases, neck pain leads to significant functional limitations in daily activities. Epidemiological data indicate a higher incidence among females [3]. The cervical facet is one of the common sites for neck pain [4].

Chronic degeneration affects the cervical vertebral bodies and intervertebral discs, leading to the formation of bone spurs and disc herniation. It also impacts the contents of the spinal canal, including the spinal cord and nerve roots, and involves degenerative changes in the facet joints [5].

Numerous studies have identified a wide range of contributing factors associated with the onset of neck pain. While a specific cause of joint dysfunction may occasionally be isolated, it is more commonly attributed to a combination of interrelated factors. The two primary predisposing factors are prolonged poor postural habits and the extent of spinal flexion during movement [6]. Additional risk factors include repetitive neck movements, prolonged computer use, sedentary lifestyle, and suboptimal ergonomic practices, especially in occupations requiring sustained static postures, such as computer professionals and drivers [7].

Cervical facet joints are involved in 56-70% of people who have chronic neck pain, and 54-60% of patients who have neck pain following whiplash injury, also referred to as zygapophysial or zygapophyseal joints, are thought to be the main cause of pain [8]. The pathophysiology of cervical facet joint syndrome (CFJS) involves degenerative, inflammatory mechanisms that contribute to localized and referred pain. The cervical facet joints (zygapophyseal joints) are synovial joints innervated by the medial branches of the dorsal rami, making them a common source of neck pain when affected by mechanical or degenerative changes. Degeneration of these joints, often due to aging, repetitive motion, or trauma, leads to cartilage thinning, subchondral sclerosis, joint space narrowing, and osteophyte formation, collectively known as facet arthropathy. These changes result in abnormal joint mechanics and loading, pain, and stiffness [9]. The joint capsule, which is richly innervated, becomes inflamed due to microtrauma or overstretching, leading to sensitization of nociceptors and the release of inflammatory mediators such as prostaglandins and substance P, further exacerbating pain. These pain signals from the cervical facet joints are transmitted via the medial branch of the dorsal ramus and often refer pain to the head, shoulder, and upper back, especially involving the C2-C3 vertebrae [10].

This study will help in gaining focus on improving the range of motion (ROM) and reducing pain and disability, which will be helpful in the improvement of movements. It is necessary to evaluate various available techniques in the treatment of CFJS. It is essential to address the complications associated with CFJS early to prevent long-term consequences. The physiotherapist typically employs various modalities, therapeutic exercises, non-thrust mobilization, and thrust manipulation as representative techniques in clinical practice to alleviate neck discomfort [11].

CFJS is a significant contributor to neck pain as well as disability in the general population. With a high prevalence and chronic nature, it is crucial to adopt an effective treatment protocol that provides lasting relief. Positional traction integrated with mobilization with movement (MWM) offers a guaranteed approach for managing this condition by improving ROM, reducing pain, and enhancing functional ability [12].

Given the growing burden of neck pain worldwide, clinicians and researchers must continue exploring innovative and evidence-based treatments to improve patient outcomes. Therefore, this study aims to emphasize the importance of improving cervical ROM and reducing pain and disability in individuals with CFJS, thereby enhancing functional mobility. Given the variety of available therapeutic approaches, it is essential to systematically evaluate their effectiveness in managing CFJS. Based on existing literature, this research will specifically focus on the integration of positional traction with MWM as a targeted intervention.

## Materials and methods

Participants

A total of 114 participants were initially enrolled in this comparative study. The study included both male and female individuals aged between 30 and 50 years with diagnosed neck pain, either radiating or non-radiating, past the shoulder for six months. Additionally, individuals with unilateral or bilateral radiating symptoms and those clinically diagnosed with CFJS by an orthopedic surgeon were included. Exclusion criteria were applied to reduce confounding variables. Individuals were excluded if they had a history of neck or spinal surgery, recent cervical trauma or fractures, congenital cervical spine disorders, cardiovascular complications, psychiatric conditions, or drug abuse. All participants were informed about the study's purpose, protocol, and interventions. Written informed consent was obtained before participation. A preliminary examination was conducted to confirm the suitability of each subject for inclusion in the study.

Study design and randomization

This was a comparative study. Participants were assigned to one of the two intervention groups using simple random sampling. The envelope method was employed for group allocation. Two sealed envelopes labeled "Group A" and "Group B" were prepared and shuffled. Each participant randomly selected one envelope immediately before beginning the intervention, determining their group. This ensured unbiased and concealed allocation. The study was single-blinded, where participants in the study were blinded. Out of 114 participants, four dropped out during the study, resulting in a final sample size of 110 (55 in each group). The study was conducted in Karad and received ethical approval from the Institutional Ethical Committee of Krishna Institute of Medical Sciences (Protocol No.: 570/2022-2023; dated: 19/05/2023). The study was carried out between 27/05/2023 and 28/05/2024.

Outcome measures

Visual Analog Scale (VAS)

It is an assessment tool designed to quantify a characteristic that exists along a continuum and is not readily measurable through direct means. The VAS is a unidimensional instrument used to assess pain intensity and has been widely applied across various adult populations. While Huskisson's (1974) [13] original study is not open access, the VAS is a widely adopted, generic tool in clinical practice and research, requiring no licensing or permission. Further validation for the use of the VAS is drawn from Delgado et al. [14].

Neck Disability Index (NDI)

The NDI is a condition-specific questionnaire consisting of 10 items that assess functional status across domains such as pain, personal care, lifting, reading, headaches, concentration, work, driving, sleeping, and recreation. Higher scores reflect greater levels of self-reported disability. The original article by Vernon and Mior (1991) is not open-access [15]. However, the NDI is publicly available for non-commercial use and has been used extensively in published clinical trials and academic research without restriction [16]. A study by John et al. provides further validation for the NDI's application in this study, indicating its classification as "non-funded academic users" [17].

Range of Motion (ROM)

Cervical ROM is prevalently used as an outcome measure in the clinical practice for people with CFJS. ROM analysis is a reliable and valid method for measuring ROM, and it is a convenient and easy outcome measure for clinical trials and physiotherapy practice [18].

Cervical ROM was assessed using a standard universal goniometer to objectively measure cervical mobility in individuals with CFJS. The assessment was conducted both before and after the intervention by the same trained physiotherapist to ensure consistency and reduce measurement variability. Participants were seated upright on a chair with feet flat on the ground and back unsupported, maintaining a neutral head and spine position. They were instructed to relax their shoulders and rest their arms on their thighs throughout the procedure. Cervical flexion and extension were measured by placing the goniometer axis over the external auditory meatus, with the stationary arm aligned perpendicular to the floor and the movable arm parallel to the base of the nose. For lateral flexion, the axis was positioned over the C7 spinous process, the stationary arm perpendicular to the floor, and the movable arm aligned with the midline of the head. Cervical rotation was assessed by placing the axis at the cranial center of the head, aligning the stationary arm with an imaginary line between the acromion processes and the movable arm with the tip of the nose. Participants were instructed to perform each motion, i.e., flexion, extension, right and left lateral flexion, and right and left rotation, within a pain-free range. Each movement was repeated three times, and the average of the three readings was recorded in degrees to ensure measurement reliability. This standardized procedure allowed for consistent and valid assessment of cervical spine mobility as an outcome measure.

Interventions

Baseline assessments were done before the initiation of any intervention. Post-treatment assessments were conducted after the four-week intervention period to evaluate changes in pain, mobility, and functional status. Each treatment session commenced with the application of a hot pack and interferential therapy (IFT) for 15 minutes in both groups as a baseline protocol. The IFT was delivered using a four-pole application with two medium-frequency currents intersecting within the target area. The carrier frequency was set at 4,000 Hz, with a beat frequency ranging between 80 and 100 Hz to achieve analgesic effects. The mode of application was continuous, as this is considered effective for subacute and chronic musculoskeletal conditions. Electrode placement followed a quadripolar arrangement: two electrodes were placed on each side of the cervical spine, at the level of the painful segment, typically from C3 to ensure proper cross-pattern current intersection. Intensity was gradually increased until the patient reported a strong but comfortable tingling sensation, without causing discomfort. Each IFT session lasted 10 minutes, and treatment was administered three times per week over four weeks.

Participants in group A received conventional physiotherapy treatment, including supervised exercises and electrotherapy. Participants in group B (experimental group) received positional traction integrated with MWM in addition to the conventional treatment provided to group A. Each participant attended three treatment sessions per week for four consecutive weeks. Additionally, they were instructed to continue the prescribed home exercise program three times weekly for three months. In group B, positional traction was administered with the participant lying prone, supported with pillows under the chest and a towel roll under the chin. The head was positioned in flexion at the edge of the plinth to target specific cervical segments. This posture was maintained for 15 seconds and was relaxed for 10 seconds, for a total duration of 10 minutes per session.

For MWM, sustained natural apophyseal glides (SNAGs) for the lower cervical spine (typically C3-C7) were used to restore pain-free ROM and relieve symptoms associated with cervical facet joint dysfunction. In this technique, the therapist applies a sustained anterior-superior glide to the spinous or articular process of the involved vertebra while the patient actively performs the restricted movement (such as rotation, flexion, or extension). The glide is maintained throughout the movement and released afterward, facilitating normal joint mechanics and reducing pain. SNAGs are typically performed in a seated position to mimic functional postures and are both safe and effective for improving cervical mobility. SNAGs can be applied for four to eight repetitions per direction, depending on patient tolerance and symptom response. Detailed intervention protocols for both groups are described in Table 1.

**Table 1:** Exercise intervention for both groups.

Duration	Group A		Group B	
	Exercise intervention	Repetitions	Exercise intervention	Repetitions
Week 1	Cervical retraction	10 sec hold × 10 reps	Cervical retraction	10 sec hold × 10 reps
	Isometric neck contractions (in all planes)	10 sec hold × 10 reps	Isometric neck contractions (in all planes)	10 sec hold × 10 reps
	Neck mobility exercises (flexion, extension, right and left lateral flexion)	10 reps	Neck mobility exercises (flexion, extension, right and left lateral flexion)	10 reps
	Shoulder shrugs	10 reps	Scapular movements: retraction and protraction	10 reps
	Scapular movements: retraction and protraction	10 reps	Mobilization with movement	6 – 10 reps per set × 2 sets
	Shoulder rotations	10 reps	Positional traction	30 – 40 sec hold × 2 sets
Week 2	Cervical retraction	10 sec hold × 15 reps	Cervical retraction	10 sec hold × 15 reps
	Isometric neck contractions (in all planes)	10 sec hold × 15 reps	Isometric neck contractions (in all planes)	10 sec hold × 15 reps
	Neck mobility exercises (flexion, extension, right and left lateral flexion)	15 reps	Neck mobility exercises (flexion, extension, right and left lateral flexion)	15 reps
	Shoulder shrugs	15 reps	Mobilization with movement	6 – 10 reps per set × 2 sets
	Scapular movements: retraction and protraction	15 reps	Scapular movements: retraction and protraction	15 reps
	Shoulder rotations	15 reps	Positional traction	30 – 40 sec hold × 2 sets
	Elevator scapulae stretch	10 sec hold × 5 reps		
	Upper trapezius stretches	10 sec hold × 5 reps		
Week 3	Cervical retraction	10 sec hold × 20 reps	Neck isometrics	10 sec hold × 20 reps
	Isometric neck contractions (in all planes)	10 sec hold × 20 reps	Chin tucks	10 sec hold × 20 reps
	Neck mobility exercises (flexion, extension, right and left lateral flexion)	20 reps	Cervical range of motion exercises	20 reps
	Shoulder shrugs	15 reps	Mobilization with movement	6 – 10 reps per set × 2 sets
	Scapular movements: retraction and protraction	15 reps	Scapular movements: retraction and protraction	15 reps
	Shoulder rotations	15 reps	Positional traction	1 min × 5- 6 set
	Elevator scapulae stretch	15 sec holds × 5 reps		
	Upper trapezius stretching	15 sec hold × 5 reps		
Week 4	Cervical retraction	10 sec hold × 25 reps	Cervical retraction	10 sec hold × 25 reps
	Isometric neck contractions (in all planes)	10 sec hold × 25 reps	Isometric neck contractions (in all planes)	10 sec hold × 20 reps
	Neck mobility exercises (flexion, extension, right and left lateral flexion)	25 reps	Neck mobility exercises (flexion, extension, right and left lateral flexion)	20 reps
	Shoulder shrugs	20 reps	Mobilization with movement	6 – 10 reps per set × 2 sets
	Scapular movements: retraction and protraction	20 reps	Scapular movements: retraction and protraction	20 reps
	Shoulder rotation	20 reps	Positional traction	1 min × 5- 6 set
	levator scapulae stretch	15 sec hold × 5 reps		
	Upper trapezius stretches	15 sec hold × 5 reps		

**Figure 1:**
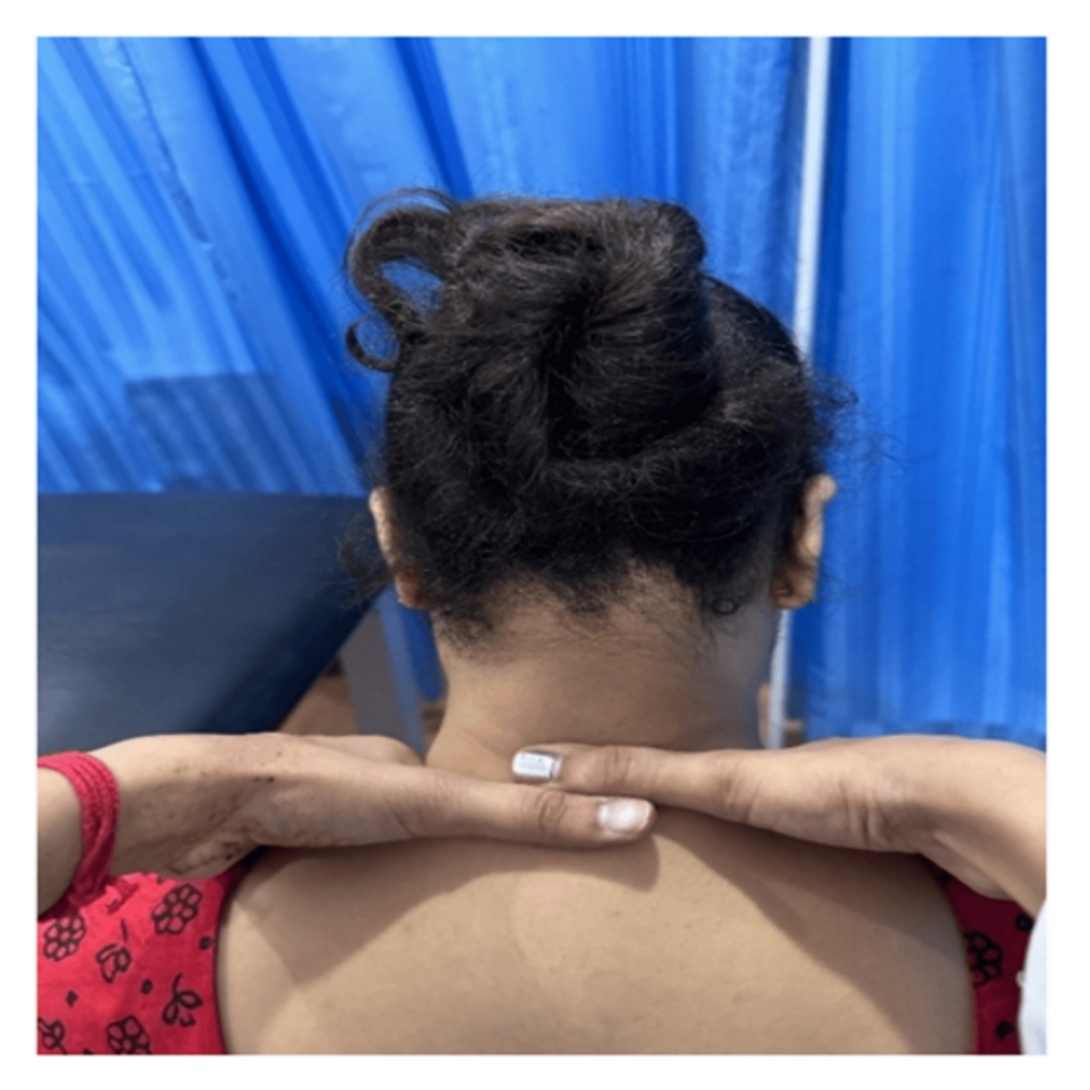
Movement with mobilization for cervical spine. Mobilization with movement (MWM) technique applied to the cervical spine during active flexion. The therapist maintains a sustained glide over the lower cervical facets while the patient performs active flexion, aiming to restore pain-free range of motion.

Figure 1 shows MWM for the cervical spine, where the therapist applies a sustained manual glide to the cervical segment while the patient performs active neck movement.

**Figure 2:**
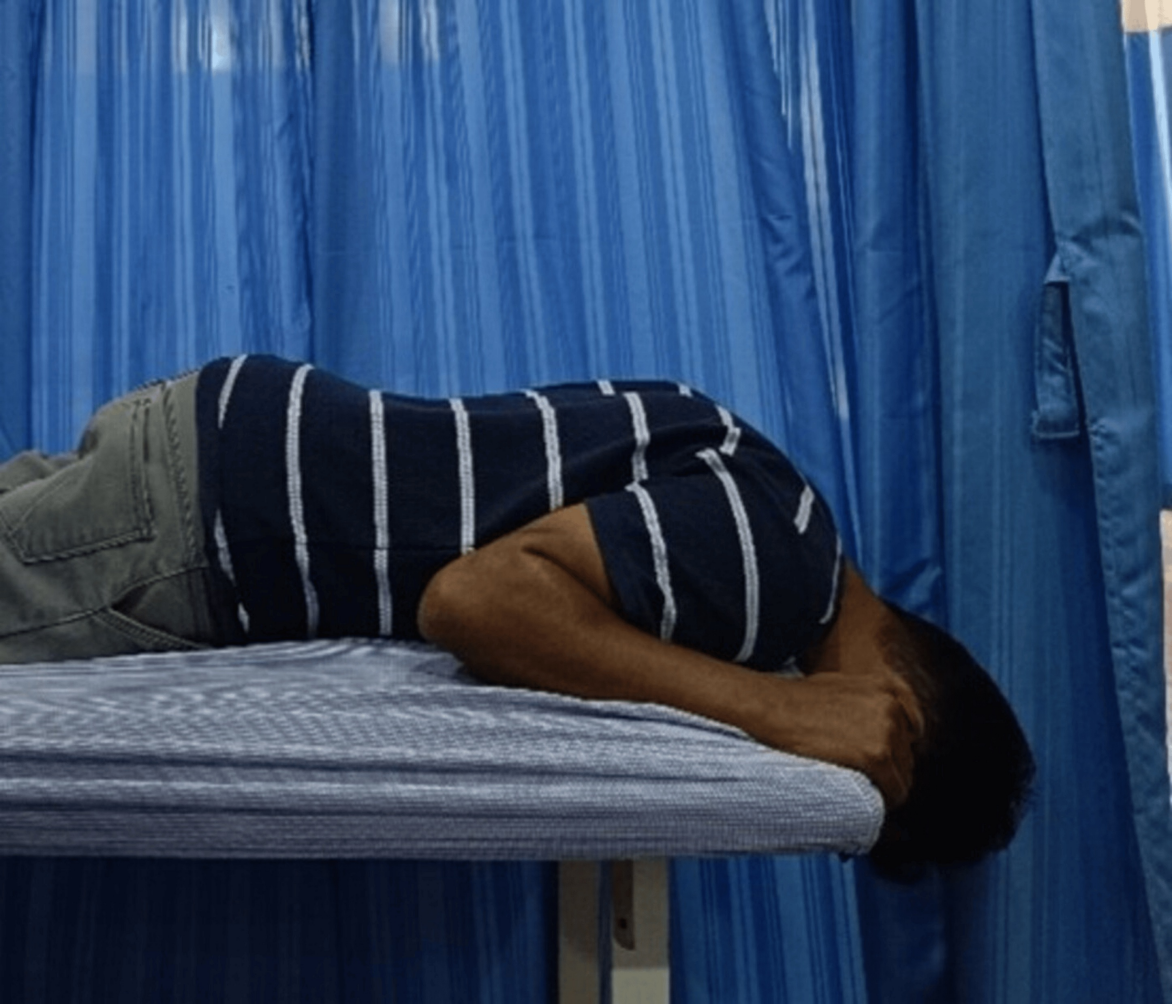
Positional traction. Positional traction for the cervical spine. The patient is positioned prone with the head rotated and supported on the forearms, allowing gravity-assisted decompression of cervical structures. This technique aims to reduce nerve root compression and relieve cervical musculoskeletal symptoms.

Figure 2 demonstrates positional traction for the cervical spine, a conservative technique used in physiotherapy to relieve pressure on the cervical nerve roots and facet joints. In this setup, the patient lies in a prone position with the head rotated and resting on the arms or a pillow, allowing gentle, gravity-assisted decompression of the cervical segments. This position helps reduce muscle tension, alleviate nerve compression symptoms such as radiating pain or tingling, and improve spinal alignment without the need for mechanical devices. It is often used for conditions like cervical radiculopathy or facet joint syndrome, where controlled, sustained stretching can help relieve symptoms. SNAGs can be applied for six to 10 repetitions per direction, depending on patient tolerance and symptom response.

To ensure adherence to the intervention protocols, all treatment sessions were conducted under the supervision of a qualified physiotherapist, allowing for real-time monitoring of technique, dosage, and progression. Participants were encouraged to continue the prescribed home exercise program three times per week for three months following the supervised sessions. To support home-based adherence, participants were provided with illustrated exercise handouts and were instructed to maintain an exercise logbook, which was reviewed during follow-up visits. Weekly telephonic reminders were also implemented to promote compliance. Attendance was recorded for all supervised sessions to track adherence to the intervention schedule. Additionally, participants were instructed to report any discomfort or adverse events immediately. No adverse events were noted during the study period, indicating both high compliance and safety of the interventions administered.

Statistical analysis

Statistical analysis of the recorded data was done. Data were entered and analyzed using SPSS version 26.0 (IBM Corp., Armonk, NY). The arithmetic mean and standard deviation were calculated for each outcome measure. The arithmetic mean was derived from adding all the values together and dividing by the total number of values. Paired t-test and unpaired t-test were used for the calculation of p-values of within-group and between-group analysis, respectively. Microsoft Excel (Microsoft Corporation, Redmond, WA) was used for drawing various graphs with given frequencies and the various percentages that were calculated with the software.

## Results

The demographic distribution of participants in groups A and B was comparable in terms of age and gender (Table 2). In the age category of 30-40 years, group A had 28 participants, while group B had 27. Similarly, in the 40-50 years category, group A had 27 participants, and group B had 28, indicating a balanced age distribution between the two groups. Regarding gender, group A comprised 29 males and 26 females, whereas group B consisted of 31 males and 24 females. This suggests that both groups were well-matched demographically, reducing the potential influence of age and gender as confounding variables in the study.

**Table 2:** Demographic status of the subjects.

Demographic variables	Category	Group A	Group B
Age	30-40 years	28	27
	40-50 years	27	28
Gender	Male	29	31
	Female	26	24

Both groups showed significant reductions in VAS scores at rest and during activity from pre- to post-test (p < 0.0001) (Table 3). Group A’s pain at rest decreased by a mean of 2.49, while group B showed a slightly greater reduction of 2.733. During the activity, group A improved by 3.29 points, and group B again showed a greater mean difference of 3.509, indicating both interventions were effective, with group B showing slightly better outcomes.

**Table 3:** Comparison of pre- and post-test mean scores of VAS (at rest and on activity) within groups. A paired t-test was used to calculate within-group analysis and p-values. VAS: visual analog scale.

VAS (at rest)	Pre-test	Post-test	Mean difference	p-value
Group A	4.98±0.61	2.49±0.78	2.49	<0.0001
Group B	4.83±0.94	2.09±0.76	2.733	<0.0001
VAS (on activity)	Pre-test	Post-test	Mean difference	p-value
Group A	6.34±0.46	3.04±1.030	3.29	<0.0001
Group B	6.09±0.57	2.58±0.85	3.509	<0.0001

The NDI scores showed a significant reduction in both groups from pre-test to post-test (p < 0.0001) (Table 4). Group A's NDI decreased from 51.40 ± 8.88 to 24.65 ± 4.47, with a mean difference of 26.74. Group B showed a greater improvement, with scores reducing from 54.16 ± 12.43 to 20.10 ± 1.73, resulting in a mean difference of 34.06. This indicates that both interventions were effective, with group B demonstrating a more substantial reduction in neck disability.

**Table 4:** Comparison of pre- and post-test mean scores of NDI within group A and group B. A paired t-test was used to calculate within-group analysis and p-values. NDI: Neck Disability Index.

NDI	Pre-test	Post-test	Mean difference	p-value
Group A	51.40±8.88	24.65±4.47	26.74	<0.0001
Group B	54.164±12.43	20.10±1.73	34.06	<0.0001

The ROM in all directions showed statistically significant improvements in both group A and group B (p < 0.05), with group B consistently demonstrating greater gains. In group A, cervical flexion improved by 0.98°, while in group B, it improved by 6.07° (Table 5). For extension, group A showed a 0.96° increase, compared to 7.49° in group B. Left and right lateral flexion improved by 0.72° and 0.52° in group A, while group B improved by 6.84° and 6.45°, respectively. Similarly, left and right rotation increased by 0.65° and 0.60° in group A, whereas group B showed greater improvements of 10.16° and 7.54°. These findings indicate that while both groups improved, group B experienced significantly greater enhancements in cervical mobility.

**Table 5:** Comparison of pre- and post-test mean scores of ROM within group A and group B. A paired t-test was used to calculate within-group analysis and p-values. ROM: range of motion.

ROM	Groups	Pre-test	Post-test	Mean difference	p-value
ROM (flexion)	Group A	28.98±3.64	29.98±3.50	-0.98	0.0038
	Group B	27.764±3.74	33.83±3.62	-6.07	<0.0001
ROM (extension)	Group A	38.16±3.81	39.12±2.25	-0.96	0.0025
	Group B	38.72±3.85	46.21±3.38	-7.49	<0.0001
ROM (left lateral flexion)	Group A	14.018±2.13	14.74±3.12	-0.72	0.0030
	Group B	13.81±2.04	20.65±1.308	-6.836	<0.0001
ROM (right lateral flexion)	Group A	14.27±2.10	14.80±2.360	-0.52	0.0030
	Group B	14.20±1.84	20.65±1.30	-6.45	<0.0001
ROM (left rotation)	Group A	38.909±3.07	39.56±3.45	-0.65	0.0011
	Group B	37.56±3.035	47.72±1.86	-10.16	<0.0001
ROM (right rotation)	Group A	39.96±2.86	40.56±2.89	-0.60	0.0015
	Group B	37.58±3.06	45.12±2.906	-7.54	<0.0001

The post-test analysis demonstrated statistically significant differences between group A and group B across all measured outcomes (Table 6). Group B exhibited lower pain intensity, as reflected by VAS scores both at rest (2.09 ± 0.76) and during activity (2.58 ± 0.85), compared to group A (2.49 ± 0.76 and 3.04 ± 1.03, respectively), with p-values of 0.0088 and 0.0118. Additionally, group B showed superior functional improvement, indicated by a lower NDI score (20.1 ± 1.73) relative to group A (24.65 ± 4.47), which was statistically significant (p < 0.0001). Furthermore, group B demonstrated greater enhancements in cervical range of motion across all directions, i.e., flexion, extension, lateral flexion, and rotation, with all p-values being <0.0001. These findings suggest that the intervention applied in group B was more effective in reducing pain, improving functional ability, and enhancing cervical mobility.

**Table 6:** Between group analysis of all outcome measures. Unpaired t-test was used to calculate between-group analysis and p-values. VAS: visual analog scale; NDI: Neck Disability Index.

Post-test values	Group A	Group B	p-value
VAS (at rest)	2.49±0.76	2.09±0.76	0.0088
VAS (on activity)	3.04±1.030	2.58±0.85	0.0118
NDI	24.65±4.47	20.1±1.73	<0.0001
Flexion	29.98±3.50	33.83±3.62	<0.0001
Extension	39.12±2.25	46.21±3.38	<0.0001
Left lateral flexion	14.74±3.12	20.65±1.308	<0.0001
Right lateral flexion	14.80±2.360	20.65±1.30	<0.0001
Left rotation	39.56±3.45	47.72±1.86	<0.0001
Right rotation	40.56±2.89	45.12±2.906	<0.0001

## Discussion

CFJS remains a significant contributor to chronic neck pain and functional limitations, particularly through restricted cervical ROM and impaired performance of daily activities. In the present study, both treatment groups, i.e., group A (conventional therapy) and group B (positional traction with MWM), showed statistically significant reductions in pain as measured by the VAS. However, group B demonstrated superior outcomes in terms of pain relief, functional improvement, and ROM enhancement, underscoring the potential advantages of integrating manual therapy techniques into clinical practice. In this study, the reduction in VAS scores in group B exceeded the minimum clinically important difference (MCID) of two points, indicating that the pain relief experienced was not only statistically significant but also meaningful to the patient in terms of daily comfort and functionality. Similarly, the improvement in NDI scores surpassed the MCID threshold of five points, suggesting a significant improvement in the participants’ ability to perform daily activities. Additionally, the observed gains in cervical ROM, particularly in flexion-extension and rotation, were well above the minimal detectable change (MDC) reported in previous literature, reflecting true improvement in joint mobility rather than measurement variability.

These findings align with the previous study, which compared isometric exercises to general exercises and found that neck isometric exercises were more effective in pain reduction. Additionally, one study conducted a meta-analysis demonstrating that isometric training significantly reduces pain scores in patients with chronic neck pain. In contrast, conventional physiotherapy methods, while effective, may not provide optimal results in cases where joint mobility remains restricted. As per the previous study, SNAGs were compared with mechanical traction and found that SNAGs were more effective in reducing pain intensity, supporting the premise that manual therapy techniques combined with positional traction may have superior analgesic effects [19].

The findings of the present study are supported by several previous investigations that emphasize the efficacy of manual therapy techniques in managing cervical spine disorders. For instance, a previous study compared SNAGs with Maitland mobilization in patients with mechanical neck pain. They reported that SNAGs were more effective in reducing pain and improving cervical ROM. In contrast, the current study employed MWM combined with positional traction, offering both dynamic joint gliding and segmental unloading. This dual mechanism likely contributed to the superior results in group B, highlighting the additional benefits of incorporating decompressive techniques alongside mobilization [20].

Similarly, another study demonstrated that mechanical traction, when added to conventional physiotherapy (including moist heat, interferential therapy, and exercises), resulted in significantly better improvements in pain and cervical mobility than conventional physiotherapy alone. Their study reinforces the mechanical value of traction in reducing neural irritation and facet joint compression. However, in contrast to their use of mechanical traction, the present study applied positional traction, which is more specific and adaptive to the patient’s comfort and spinal alignment. When integrated with MWM, it offers a more comprehensive and individualized treatment approach, which explains the greater improvements seen in group B of this study [21].

A plausible explanation for the superior outcomes in group B lies in the mechanisms of MWM and positional traction. MWM, developed by Mulligan, involves sustained joint gliding during movement, reducing pain and improving function [20]. When combined with positional traction, which decompresses the facet joints and reduces inflammation, it likely contributes to better restoration of movement.

Limitations, strengths, and recommendations

This study had some limitations, including the heterogeneous occupational backgrounds of the participants, which may have influenced the outcomes due to varying physical demands. Also, the sample size was relatively small, which may limit the generalizability of the findings. Additionally, the experimental intervention involved a combination of positional traction and MWM, making it difficult to isolate the individual contribution of each component. Another limitation of this study is the absence of a placebo or sham intervention group, which restricts the ability to control for non-specific treatment effects such as therapist attention or patient expectations. A larger sample size would improve the generalizability of the findings. However, the study had notable strengths, that is, the use of a randomized sampling technique to ensure unbiased allocation and the application of standardized outcome measures like VAS, ROM, and NDI, which enhanced the reliability of the results. The integration of positional traction with MWM provided a novel treatment approach, contributing to advancements in physiotherapy research and clinical practice. Future research studies should consider including a larger, more homogenous sample and conducting long-term follow-ups to assess the sustained effects of the intervention. Further research can also explore the combination of positional traction and mobilization with other physiotherapy techniques to optimize treatment outcomes for individuals with CFJS.

## Conclusions

The findings of the present study suggest that the combination of positional traction and MWM is superior in the management of individuals diagnosed with CFJS. The findings of the present study showed that on comparing group A and group B, the experimental group (group B) showed superior improvements in relieving pain and disability and improving cervical ROM in individuals with CFJS. These findings suggest the potential value of integrating positional traction and MWM into clinical physiotherapy protocols for managing cervical facet joint dysfunction. The improvements observed in both subjective and objective outcome measures highlight the potential for this combined intervention to enhance functional recovery, reduce reliance on pharmacological management, and improve overall quality of life in affected individuals. Future studies should be conducted with larger sample sizes and extended follow-up periods to validate the long-term effectiveness and sustainability of these treatment outcomes. Additionally, incorporating objective outcome measures such as imaging or electromyography could further enhance the understanding of underlying physiological changes.

## References

[1] Yang J, Yang M, Lin Q, Fu J, Xi R (2022). Effects of isometric training on the treatment of patients with neck pain: a meta-analysis. Medicine (Baltimore).

[2] Gellhorn AC (2011). Cervical facet-mediated pain. Phys Med Rehabil Clin N Am.

[3] van Rooijen DE, Lalli S, Marinus J (2015). Reliability and validity of the range of motion scale (ROMS) in patients with abnormal postures. Pain Med.

[4] Boonstra AM, Schiphorst Preuper HR, Reneman MF, Posthumus JB, Stewart RE (2008). Reliability and validity of the visual analogue scale for disability in patients with chronic musculoskeletal pain. Int J Rehabil Res.

[5] Pragassame SA, Mohandas Kurup VK, Kour J (2020). Efficacy of sustained natural apophyseal glides Mulligan technique on mobility and function in patients with cervical spondylosis: an experimental study. J Nat Sci Biol Med.

[6] Young BA, Walker MJ, Strunce JB, Boyles RE, Whitman JM, Childs JD (2009). Responsiveness of the Neck Disability Index in patients with mechanical neck disorders. Spine J.

[7] Wong JJ, Shearer HM, Mior S (2016). Are manual therapies, passive physical modalities, or acupuncture effective for the management of patients with whiplash-associated disorders or neck pain and associated disorders? An update of the Bone and Joint Decade Task Force on Neck Pain and Its Associated Disorders by the OPTIMa Collaboration. Spine J.

[8] Hurley RW, Adams MCB, Barad M (2021). Consensus practice guidelines on interventions for cervical spine (facet) joint pain from a multispecialty international working group. Pain Med.

[9] Yoganandan N, Stemper BD, Baisden JL, Pintar FA, Paskoff GR, Shender BS (2015). Effects of acceleration level on lumbar spine injuries in military populations. Spine J.

[10] Chen C, Lu Y, Kallakuri S, Patwardhan A, Cavanaugh JM (2006). Distribution of A-δ and C-fiber receptors in the cervical facet joint capsule and their response to stretch. J Bone Joint Surg Am.

[11] Hoy DG, Protani M, De R, Buchbinder R (2010). The epidemiology of neck pain. Best Pract Res Clin Rheumatol.

[12] Kirpalani D, Mitra R (2008). Cervical facet joint dysfunction: a review. Arch Phys Med Rehabil.

[13] Huskisson EC (1974). Measurement of pain. Lancet.

[14] Delgado DA, Lambert BS, Boutris N, McCulloch PC, Robbins AB, Moreno MR, Harris JD (2018). Validation of digital visual analog scale pain scoring with a traditional paper-based visual analog scale in adults. J Am Acad Orthop Surg Glob Res Rev.

[15] Vernon H, Mior S (1991). The Neck Disability Index: a study of reliability and validity. J Manipulative Physiol Ther.

[16] (2025). Mapi Research Trust. Neck Disability Index (NDI). https://eprovide.mapi-trust.org/instruments/neck-disability-index#contact_and_conditions_of_use.

[17] John B, Røe C, Brox JI, Sveinall H, Ignatius J, Wilhelmsen M, Skatteboe S (2025). Responsiveness and minimal important change of neck disability index and numeric pain rating scale for neck patients in the Norwegian neck and back register. Eur Spine J.

[18] Araujo GG, Pontes-Silva A, Leal PD (2024). Goniometry and fleximetry measurements to assess cervical range of motion in individuals with chronic neck pain: a validity and reliability study. BMC Musculoskelet Disord.

[19] Kanlayanaphotporn R, Chiradejnant A, Vachalathiti R (2009). The immediate effects of mobilization technique on pain and range of motion in patients presenting with unilateral neck pain: a randomized controlled trial. Arch Phys Med Rehabil.

[20] Prayerna B, Subbiah K, Asser PA, Milanese S (2019). Effectiveness of Mulligan’s sustained natural apophyseal glide (SNAG) over first rib in reducing pain and improving cervical rotation in individuals with mechanical neck dysfunction. J Clin of Diagn Res.

[21] Colombo C, Salvioli S, Gianola S, Castellini G, Testa M (2020). Traction therapy for cervical radicular syndrome is statistically significant but not clinically relevant for pain relief. A systematic literature review with meta-analysis and trial sequential analysis. J Clin Med.

